# DIFFERENCES IN ADVANCED CARE PLANNING BY LEVEL OF COGNITIVE IMPAIRMENT

**DOI:** 10.1093/geroni/igae098.3312

**Published:** 2024-12-31

**Authors:** Isabella Arellanes, Mireille Jacobson

**Affiliations:** University of Southern California Leonard Davis School of Gerontology, Los Angeles, California, United States; University of Southern California, Los Angeles, California, United States

## Abstract

Advance Care Planning (ACP) allows patients to make decisions regarding their health care while they still have the ability to do so. ACP may be particularly important for individuals experiencing cognitive decline who are likely to become unable to make decisions for themselves. This study identifies differences in advance care planning based on cognitive impairment status. This sample includes 6,650 older adults between the age of 65-90 years from the 2018 wave of the Health and Retirement Study. Cognition was categorized based on the Langa-Weir cognition classifications, with normal cognition classified by scores of 12-27 (n= 5,313), “cognitive impairment, not dementia” by scores of 7-11 (n= 1,085), and dementia by scores between 0-6 (n= 252). Among respondents with a normal cognition score, 65% reported having either a living will or power of attorney. Among those categorized as having “cognitive impairment, not dementia” and dementia, the percentages of ACP completion were lower, 59% and 54%, respectively. In a regression model controlling for race/ethnicity, gender, years of education, marital status, AD or dementia diagnosis, income, and healthcare utilization, all but diagnosis were significant predictors of ACP. There was a significant difference in ACP between those with normal cognition as those classified as having “cognitive impairment, not dementia” (p< 0.05). Compared to those in the normal cognition group, those in the “cognitive impairment, not dementia” group are 1.3 times more likely to engage in ACP (p < 0.05). These findings suggest differences in significant predictors of ACP based on level of cognitive impairment.

